# Adipose-Derived Stem Cells from Fat Tissue of Breast Cancer Microenvironment Present Altered Adipogenic Differentiation Capabilities

**DOI:** 10.1155/2019/1480314

**Published:** 2019-08-14

**Authors:** Federica Rey, Elena Lesma, Daniela Massihnia, Emilio Ciusani, Sara Nava, Chiara Vasco, Ghina Al Haj, Giorgio Ghilardi, Enrico Opocher, Alfredo Gorio, Stephana Carelli, Anna Maria Di Giulio

**Affiliations:** ^1^Laboratory of Pharmacology, Department of Health Sciences, University of Milan, Via A. di Rudinì 8, 20142 Milan, Italy; ^2^Pediatric Clinical Research Center “Fondazione Romeo e Enrica Invernizzi”, University of Milan, 20142 Milan, Italy; ^3^Laboratory of Clinical Pathology and Medical Genetic, Fondazione IRCCS Neurological Institute C. Besta, Milan, Italy; ^4^Cell Therapy Production Unit, Laboratory of Cellular Neurobiology, Cerebrovascular Unit, and Unit of Molecular Neuro-Oncology, Fondazione IRCCS Istituto Neurologico Carlo Besta, Milan, Italy; ^5^Department of Health Sciences, San Paolo Hospital, University of Milan, Milan, Italy; ^6^General Surgery Unit, Department of Health Sciences, University of Milan, Via A. di Rudinì 8, 20142 Milan, Italy

## Abstract

Mesenchymal stem cells (MSCs) are multipotent cells able to differentiate into multiple cell types, including adipocytes, osteoblasts, and chondrocytes. The role of adipose-derived stem cells (ADSCs) in cancers is significantly relevant. They seem to be involved in the promotion of tumour development and progression and relapse processes. For this reason, investigating the effects of breast cancer microenvironment on ADSCs is of high importance in order to understand the relationship between tumour cells and the surrounding stromal cells. With the current study, we aimed to investigate the specific characteristics of human ADSCs isolated from the adipose tissue of breast tumour patients. We compared ADSCs obtained from periumbilical fat (PF) of controls with ADSCs obtained from adipose tissue of breast cancer- (BC-) bearing patients. We analysed the surface antigens and the adipogenic differentiation ability of both ADSC populations. C/EBP*δ* expression was increased in PF and BC ADSCs induced to differentiate compared to the control while PPAR*γ* and FABP4 expressions were enhanced only in PF ADSCs. Conversely, adiponectin expression was reduced in PF-differentiated ADSCs while it was slightly increased in differentiated BC ADSCs. By means of Oil Red O staining, we further observed an impaired differentiation capability of BC ADSCs. To investigate this aspect more in depth, we evaluated the effect of selective PPAR*γ* activation and nutritional supplementation on the differentiation efficiency of BC ADSCs, noting that it was only with a strong differentiation stimuli that the process took place. Furthermore, we observed no response in BC ADSCs to the PPAR*γ* inhibitor T0070907, showing an impaired activation of this receptor in adipose cells surrounding the breast cancer microenvironment. In conclusion, our study shows an impaired adipogenic differentiation capability in BC ADSCs. This suggests that the tumour microenvironment plays a key role in the modulation of the adipose microenvironment located in the surrounding tissue.

## 1. Introduction

Mesenchymal stem cells (MSCs) are used in regenerative medicine for the treatment of tissue damage after specific pathological processes, such as graft versus host disease, articular cartilage, and other bone injuries [1]. MSCs can be efficiently derived from different tissues such as the bone marrow, the adipose tissue, the skin, and the muscle [2]. They are multipotent cells with the ability to differentiate into various cell types, such as adipocytes, osteoblasts, and chondrocytes [3]. The adipose tissue is an important endocrine and metabolic organ characterized by different functions, ranging from hormone secretion to heat production. It contains different cell types such as stromal vascular cells, which include adipose stem cells (ADSCs), and endothelial progenitor cells [4]. ADSCs have proangiogenic, antiapoptotic, anti-inflammatory, and immunomodulatory effects, through paracrine secretion of cytokines, chemokines, and growth factors [5, 6]. These functions make them optimal candidates for cellular therapy in regenerative medicine [7]. Even so, ADSCs are also involved in promoting tumour development and progression, as well as relapse processes in different cancer types [8]. Recent studies focused on the interaction between the stromal resident cells, such as ADSCs, cancer-associated fibroblasts, and cells deriving from primary tumour [9]. Several evidences indicate that the cellular functions associated with invasion and metastasis are not produced by carcinoma cells, but they are a transient response to signals that tumour cells receive from their stromal microenvironment [10, 11]. Indeed, human breast cancer cells mixed with bone marrow-derived human MSCs, injected subcutaneously, in a mouse model recruit murine MSCs and the further circulating human cancer cells, also stimulating the *de novo* secretion of the chemokine CCL5. This suggests that invasion and metastasis of cancer cells from the primary site are driven by signals released from the stroma of the primary tumour [10]. Indeed, the interaction of human breast carcinoma cells with bone marrow-derived human MSCs significantly increases metastatic potency. Moreover, the interaction of cancer cells and MSCs induces the transformation of MSCs into cancer-associated fibroblasts through the production of CCL5 and osteopontin, promoting tumour progression [12].

For these reasons, investigating the effects of breast cancer (BC) microenvironment on ADSCs could be of crucial importance in order to understand the interaction between the tumour and its microenvironment. Indeed, it is known that stromal cells located near the BC microenvironment may develop into mammary carcinogenesis [13]. Interestingly, even if the adipose tissue is the most abundant stromal constituent in the breast, little is known about the involvement of resident ADSCs in the BC development. BC is a frequent carcinoma in postmenopausal women [14] and is classified in different groups based on the gene expression profile: luminal A, luminal B, HER2, and basal-like breast cancer (BLBC) [15]. Increased adiposity and obesity are associated with an elevated risk of the onset of the most prevalent form of BC [16]. Clinical experiences have sustained that BC often develops in close association with fat [9]; moreover, age is a risk factor, since during the aging process the mammary tissue becomes richer in fat and less dense [17]. Adipose tissue can be easily isolated in abundant quantities from many sites, such as the abdomen and breast [18]. The adipogenic process conducted by ADSCs is subsequent to the activation of PPAR*γ*, the receptor responsible for the induction of this process [19]. PPAR*γ*'s expression has been reported to be deregulated in breast cancer patients, along with other cancer types [20, 21]. Furthermore, multiple studies are investigating the therapeutic potential of PPAR*γ* modulators as innovative cancer therapy [22, 23]. The role that nutrition and especially low- versus high-fat diets play in breast cancer outcomes becomes even more highlighted [24–28]. Indeed, studies demonstrated that a low-fat diet is associated with a significant improvement in overall survival [24]. Multiple protocols use lipid mixtures in *in vitro* cultures to mimic a high-fat diet and investigate cellular responses [29]. Moreover, in murine experimental models, it has been demonstrated that obesity is associated with increased survival and persistence of residual tumour cells [26]. Furthermore, dietary fat could modulate the homeostasis of the adipose tissue acting on processes such as autophagy and apoptosis [30].

The aim of our study was to investigate whether ADSCs isolated from the adipose tissue of BC-bearing patients have specific cellular and functional characteristics that may be influenced, in a bidirectional manner, by the BC microenvironment and by the tumour itself. Indeed, there could be differences in qualitative and quantitative characteristics of these ADSC populations, in terms of their adaptation to extended culture and multipotency, or their response to specific stimuli, which ultimately relates to practical considerations regarding also their possible clinical use.

## 2. Materials and Methods

### 2.1. Subjects

Tissues were isolated within the Surgery Unit of San Paolo Hospital. Patients gave their informed consent according to the Declaration of Helsinki. The study was approved by the Institutional Review Board of Milan of San Paolo Hospital (n. 11698; July 06, 2016). The study participants were 20 female patients with breast cancer (BC; Table 1) and 8 women who underwent surgery for the removal of umbilical hernia or epigastric hernia (CTRL; periumbilical fat (PF)). All patients enrolled in this study had a normal BMI (18.5 ≤ BMI ≤ 24.9). In BC patients, a sample of breast fat was taken 5 cm away from the cancer lesion. Control fat samples were collected from the subcutaneous tissue of the abdomen of patients affected with elective benign, noninflammatory, and noninfectious diseases, namely, umbilical and epigastric hernias. Control subjects and BC carriers belonged to the same age group (median 62.3 controls and 63.5 years BC). Subcutaneous abdominal adipose tissue is rich in stem cells and is commonly used for breast lipofilling for its affinity to breast fat.

### 2.2. Primary Cell Culture

Primary cell cultures, obtained from PF and from mammary fat of patients with BC, were cultured following previously published protocols [31]. The cells were cultured in Minimum Essential Media *α* medium (MEM*α*; Invitrogen, Carlsbad, CA, USA) supplemented with 15% Fetal Bovine Serum (FBS; JRH Bioscience, Lenexa, KS, USA) and incubated at 37°C in 5% CO_2_. After 15 days, the medium and the tissues were removed and adherent cells were maintained in culture. The medium was changed every 3 days until the cells reached confluence (85%). To avoid spontaneous differentiation, cells were maintained at a subconfluent culture level. When the cells reached 85% confluence, they were detached with 0.05% trypsin/EDTA solution (Thermo Fisher), collected by centrifugation (1200 rpm × 5 min) and expanded in culture or cryopreserved at −80°C in the presence of dimethyl sulfoxide (DMSO; 10%, FBS 90%). All *in vitro* experiments were performed in 5 isolates of each group (PF ADSCs and BC ADSCs) with similar results. All reported images are representatives of what was observed in the 5 isolates. Immunofluorescence and Oil Red O quantifications were determined as the mean of 3 fields/isolate for 5 isolates.

### 2.3. Proliferation

Cellular proliferation was analysed by cumulative population doubling (CPD). Cells were plated at a density of 7000 cells/cm^2^ and counted by using trypan blue (Life Technologies, Gaithersburg, MD) at 85% confluence. Curves were obtained calculating the population doubling (PD) with the following:

(1) $$\begin{array}{c} \text{PD}=\frac{\left[\log_{10}\text{NH}-\log_{10}\text{NS}\right]}{\log_{10}2}, \end{array}$$

where NS is the cell number at seeding (7000 cells/cm^2^) and NH is the cell number at harvest. To calculate the CPD, the PD determined for each passage is then added to the CPD of the previous passage. Cells were incubated for 5 min with 0.1% trypan blue (Life Technologies, Gaithersburg, MD), examined by light microscopy with a minimum of 100 total cells counted per slide, and scored as able (live) or unable to exclude the dye (apoptotic).

### 2.4. Immunophenotypic Characterization

Cultures of PF ADSCs and BC ADSCs at different passages (lower than passage 4) were phenotypically characterized following reference guidelines [32, 33]. ADSCs obtained from PF- or BC-bearing patients were detached with 0.05% trypsin/EDTA (Thermo Fisher), washed with PBS, and 100000 cells were resuspended in 250 *μ*L of PBS without Ca^2+^ and Mg^+^ (Euroclone, Pero, Italy) and incubated with antibodies directed against specific surface markers. Cells were incubated on ice for 30 minutes with antibodies anti-CD44 (BD Biosciences, San Jose, CA), anti-CD90 (Millipore, Massachusetts, USA), anti-CD34 (Miltenyi Biotec, Calderara di Reno, BO, Italy), anti-CD45 (BD Biosciences), anti-CD146 (Biocytex, USA), anti-CD31 (Miltenyi Biotec), anti-CD56 (Miltenyi Biotec), anti-CD105 (Serotec, Bio-Rad, Segrate, MI, Italy), anti-CD144 (R&D Systems, Minneapolis, MN, USA), anti-CD166 (BD Biosciences), anti-CD133/2 (Miltenyi Biotec), anti-CD73 (BD Biosciences), and anti-vascular endothelial growth factor 2 (VEGFR2; R&D Systems). Cells were pelleted, washed, and fixed in 4% paraformaldehyde (Sigma-Aldrich) for 20 minutes. Fluorescence-activated cell sorting (FACS) analysis was performed on a FACSVerse flow cytometer (BD Biosciences), equipped with the Cell Sweet software for data analysis.

### 2.5. Immunofluorescence Staining

After seeding and differentiation (3500 cells/cm^2^) onto glass slides, cells were grown until 85% confluence. They were then fixed with 4% paraformaldehyde (Sigma-Aldrich). After saturation and permeabilization (4% BSA (Sigma-Aldrich), 0.3% Triton X-100 (VWR International, Radnor, PA, USA)), cells were incubated overnight at 4°C with primary antibodies against PPAR*γ* (1 : 100; Cell Signaling Technology, Beverly, MA, USA), C/EBP*β* (1 : 100; Cell Signaling Technology), C/EBP*δ* (1 : 100 Santa Cruz, Dallas, TX, USA), FABP4 (1 : 100 Cell Signaling Technology), leptin R (1 : 100; Abcam, Cambridge, UK), and adiponectin (1 : 100 Abcam). Cells were rinsed and then probed for 45 minutes with secondary antibody Alexa Fluor 488 (Invitrogen, Carlsbad, CA, USA). Nuclei were counterstained with DAPI (2 *μ*g/mL in PBS; Sigma-Aldrich), and glasses were mounted with FluorSave™ (Millipore). Images were taken using a Leica SP2 confocal microscope with He/Kr and Ar lasers (Heidelberg, Germany). In negative control experiments, primary antibodies were replaced with equivalent concentrations of unrelated IgG of the same subclass. The quantification of positive cells was performed by considering a minimum of nine independent fields (three fields/three coverslips/treatment) captured with a 20x objective. The number of positive cells was expressed as the percentage to the total cell number given by DAPI nuclear staining.

### 2.6. Adipogenic Differentiation and Oil Red O Staining

PF and BC ADSCs were seeded (6000 cells/cm^2^) in adipogenic medium consisting of DMEM high glucose (Euroclone, MI, Italy) supplemented with 10% FBS, 1 *μ*mol/L dexamethasone (Sigma-Aldrich, St. Louis, MO, USA), 0.5 mM 3-isobutyl-1-methyl-xanthine (Sigma-Aldrich), and 10 *μ*M insulin (Sigma-Aldrich) [34, 35]. Alternatively, BC ADSCs were also differentiated with standard adipogenic medium supplemented with 1 *μ*g/mL troglitazone (Sigma-Aldrich), a potent PPAR*γ* activator [36, 37], or with a 10% lipid mixture (Sigma-Aldrich) in order to mimic a high-fat diet [29]. The final lipidic concentration in this last reagent was thus 200 ng/mL arachidonic acid; 1 *μ*g/mL linoleic, linolenic, myristic, oleic, palmitic, and stearic acids; 22 *μ*g/mL cholesterol; and 7 *μ*g/mL tocopherol acetate. The differentiation potential was assessed also after the supplementation with the potent and selective PPAR*γ* antagonist T0070907 (1 *μ*M) [38]. After 7 days in culture, a sufficient time for adipocyte differentiation and lipid droplet formation [39–41], cells were fixed in 4% formaldehyde for 1 h and stained with Oil Red O (Sigma-Aldrich). Total counts of positive cells were performed, and the number of positive cells was expressed as the percentage to the total cells. To quantify the intracellular lipid accumulation of Oil Red O, the stained lipid droplets were eluted with 100% isopropanol for 10 min. The optical density was measured at 520 nm by a spectrophotometer [41]. These evaluations were performed by means of the software ImageJ (NIH).

### 2.7. RNA Extraction and Real-Time PCR

For gene expression analysis, cells were plated at a density of 6000 cells/cm^2^ and induced to differentiate for 7 days. Total RNA was extracted using a TRIZOL® reagent (Life Technologies) following the manufacturer's instructions and then quantified (NanoPhotometer® NP80, Implen). Total RNA (1 *μ*g) was reverse transcribed using an iScript cDNA synthesis kit (Bio-Rad) according to the manufacturer's instructions. Real-Time PCR was performed with the StepOnePlus™ Real-Time RT-PCR System (Thermo Fisher) using iQ SYBR Green Supermix (Bio-Rad). Primers were designed using the NCBI's Primer-BLAST and are listed in Supplementary Table 1. Gene expression was calculated using the 2^-*ΔΔ*Ct^ method, and GAPDH was used as a housekeeping gene.

### 2.8. Statistics

Results are expressed as the means ± SD and analysed using GraphPad Prism software (version 5.0, GraphPad Software, San Diego, California). Two-tailed unpaired Student's *t*-test was used to analyse normally distributed data. When three or more value sets were compared, one-way ANOVA was used followed by Tukey's posttest applied. The statistical significance was accepted for a *p* value < 0.05.

## 3. Results

### 3.1. Proliferation

**Figure 1:**
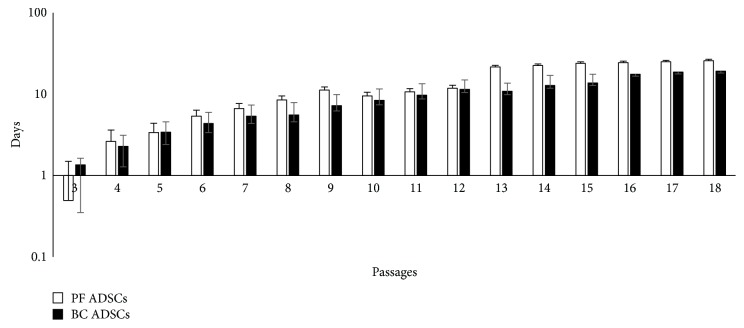
Long-term multipassage cultures of BC ADSCs and PF ADSCs. The graph shows a cumulative population doubling (CPD) across multiple consecutive passages (mean ± SD; *n* = 5). The cells were plated at a density of 7000 cell/cm^2^ and counted in triplicate by trypan blue exclusion at 85% confluence.

BC ADSC and PF ADSC proliferation was studied with cumulative population doubling (CPD) curves (Figure 1). The cellular proliferative activity gradually increased with the progression in the number of passages reaching a plateau at around passage 13 in PF ADSCs and around passage 16 in BC ADSCs. This indicates that BC and PF ADSCs have a similar proliferating activity.

### 3.2. Phenotypic Characterization of PF and BC ADSCs

**Figure 2:**
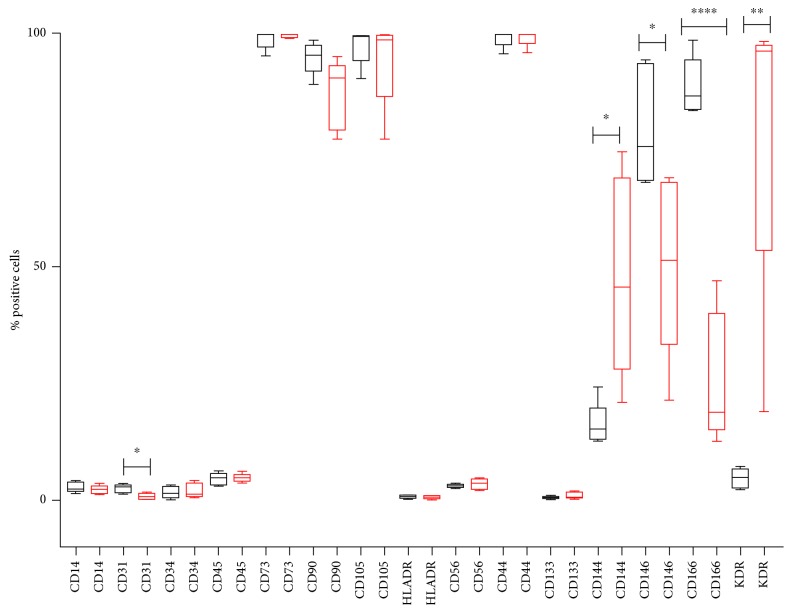
Cell surface phenotype of 5 PF ADSCs (black boxes) and 5 BC ADSCs (red boxes). All percentages were obtained by flow cytometry analysis. Results were obtained from three independent experiments for each isolate. The statistical significance was determined by Student's *t*-test; ^∗∗∗∗^ *p* < 0.0001, ^∗∗^ *p* < 0.01, and ^∗^ *p* < 0.05 vs. PF ADSCs.

To evaluate the phenotypic features of the primary cultures, flow cytometry analysis of the two cellular populations was performed. We evaluated the expression of mesenchymal surface markers (CD73, CD90, and CD105), hematopoietic markers (CD14, CD45, CD56, and CD133), D-related human leukocyte antigen (HLA-DR), endothelial markers (CD31 and CD34), adhesion surface markers (CD144, CD44, CD146, and CD166), and receptor for vascular endothelial growth factor 2 (VEGFR2 also known as KDR). The evaluation was performed in triplicate at passage 4 for all investigated isolates (Supplementary Figure 1).  Figure 2 shows the characterization of surface markers restricted to five isolates for each group, PF and BC ADSCs, which were used for all the *in vitro* experiments. PF and BC ADSCs strongly express the mesenchymal markers CD73 and CD105, with similar levels (close to 100%), and CD90, which is slightly less present in BC ADSCs. Few cells in both groups were positive to the hematopoietic markers CD14, CD45, and CD56; to the endothelial markers CD31 and CD34; and to HLA-DR and CD133. The percentage of cells positive to CD44 was very high (close to 100%), in both cell types. The adhesion surface marker CD144 and the receptor for vascular endothelial growth factor 2 (KDR) were significantly more expressed in BC ADSCs. On the contrary, CD146 and CD166 (adhesion surface markers) were significantly more expressed in PF ADSCs than BC ADSCs.

### 3.3. Expression of Adipogenic Markers in BC and PF ADSCs

**Figure 3:**
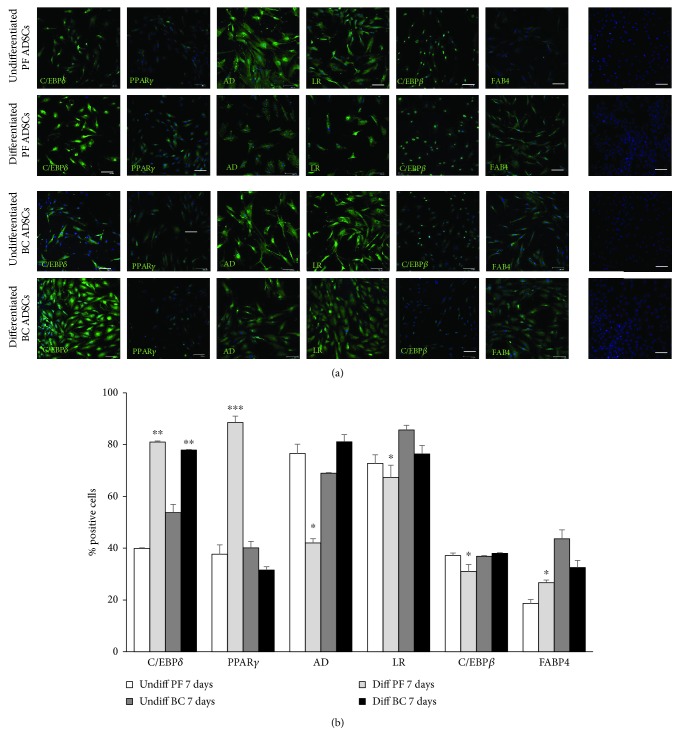
Immunofluorescence analysis of ADSC marker expression after 7 days of culture in standard medium (undifferentiated ADSCs) and in adipogenic differentiation medium (differentiated ADSCs). (a) Immunofluorescence assay for C/EBP*δ*, PPAR*γ*, adiponectin (AD), leptin receptor (LR), C/EBP*β*, and FAB4 (green labelling) which are markers of adipogenesis progression. Nuclei were stained with DAPI (blue labelling). Negative control isotype staining was performed using normal goat serum in place of the primary antibody. Scale bars: 50 *μ*m. (b) Quantification of cell positivity to adipogenic markers. The number of positive cells, expressed as the percentage to the total cell number given by DAPI nuclear staining, was calculated as an average of 15 different fields for each marker (3 fields/isolate). Error bars represent the SEM for three experiments. The statistical significance was determined by Student's *t*-test; ^∗^ *p* < 0.05 vs. undifferentiated ADSCs; ^∗∗^ *p* < 0.01 vs. undifferentiated ADSCs; ^∗∗∗^ *p* < 0.001 vs. undifferentiated ADSCs.

PF and BC ADSCs were induced to differentiate with adipogenic medium for 7 days and then compared to nondifferentiated cells (cells grown in standard maintenance medium). After 7 days of adipogenic induction, the expression of differentiation markers was investigated by immunofluorescence with specific antibodies. A statistically significant increase of C/EBP*δ* expression, a marker of preadipocyte stages, was observed in both PF and BC ADSCs induced to differentiate compared to cells grown in control medium (Figures 3(a) and 3(b)). Localization of C/EBP*δ* was greatly increased in the nuclei of both differentiated ADSCs. Adipogenic differentiation caused the increase of PPAR*γ* expression, an active modulator of lipid metabolism, only in PF ADSCs. The levels of this factor in BC under adipogenic stimulation were comparable to control standard culture conditions (undifferentiated cells). The expression of adiponectin was reduced in PF-differentiated ADSCs while it was slightly increased in BC ADSCs induced to differentiate. Leptin receptor and C/EBP*β* expression in PF-differentiated ADSCs was decreased compared to the same cells maintained in control conditions. No significant variation was found for the leptin receptor and C/EBP*β* in BC ADSCs induced to differentiate compared to control BC ADSCs. The levels of FABP4, a regulator of fatty acid, increased in differentiated PF ADSCs whereas its expression decreased in differentiated BC ADSCs. Together, these results suggest an impairment in adipogenic differentiation capabilities of BC ADSCs compared to PF ADSCs.

**Figure 4:**
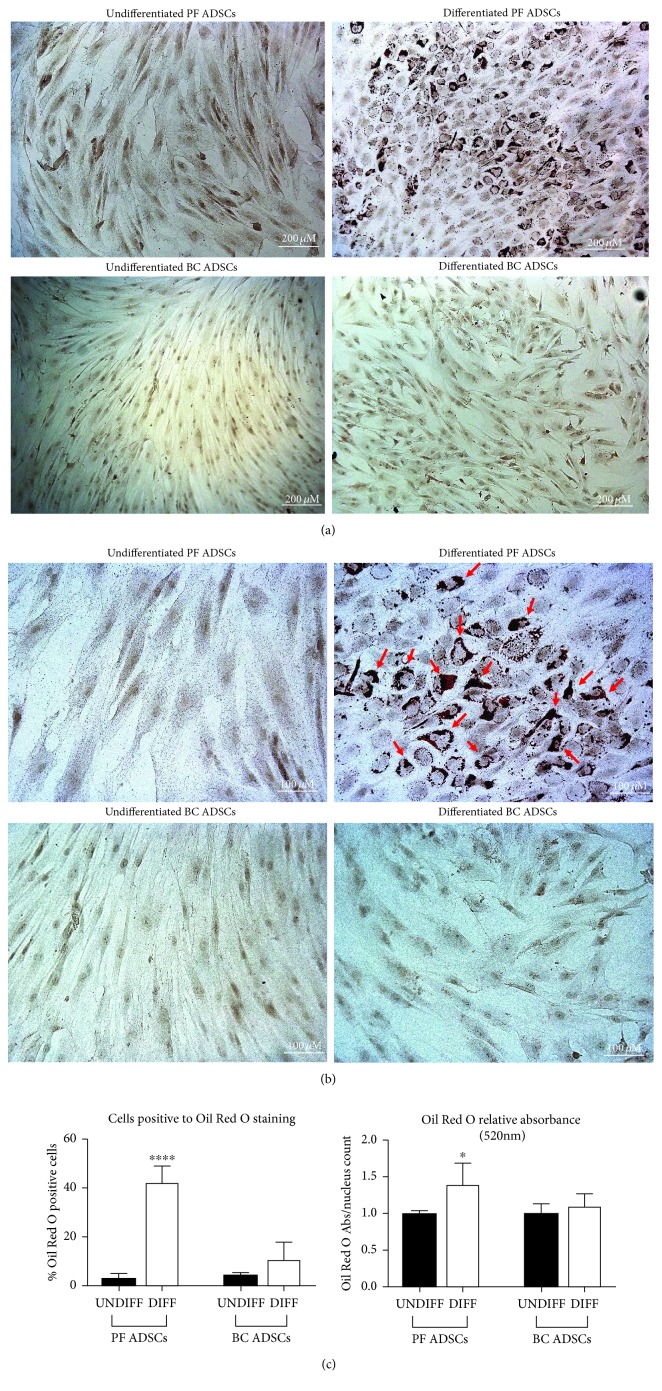
Adipogenesis was revealed by Oil Red O staining for lipid droplet accumulation. (a) Representative images of PF ADSCs and BC ADSCs in control conditions and after adipogenic differentiation for 7 days (differentiated ADSCs). Scale bars: 200 *μ*m. Data are representatives of five different isolates. (b) Magnifications of (a) images of PF ADSCs and BC ADSCs in control conditions and after adipogenic differentiation for 7 days (differentiated ADSCs). Accumulation of lipid droplets, stained with Oil Red O, is highlighted with red arrows. Scale bars: 100 *μ*m. Data are representatives of five different isolates. (c) Percentage of cells positive to Oil Red O staining and quantification of Oil Red O extracted from lipid droplets measured at 520 nm normalized over nucleus counts. Data are reported as the mean ± SD of 5 different isolates. The statistical significance was determined by Student's *t*-test; ^∗∗∗∗^ *p* < 0.0001 and ^∗^ *p* < 0.05 vs. UNDIFF PF ADSCs.

To investigate further the possible impairment of BC ADSC adipogenesis, the efficiency of the process was evaluated with Oil Red O staining in order to examine the lipid droplet formation. Indeed, Oil Red O staining (red colour) accumulated in lipid droplets in PF ADSCs, indicating a successful differentiation (Figures 4(a) and 4(b)). The quantification of both Oil Red O-positive cells and optical density after its extraction was analysed. As expected, PF ADSCs differentiated and presented a higher lipid accumulation compared to the control (Figure 4). More than 45% of differentiated PF ADSCs resulted positive to the Oil Red O staining. The quantification of lipid accumulation obtained by measuring the absorbance at 520 nm after the extraction procedure confirmed these observations. Interestingly, the BC ADSCs induced to differentiate did not present lipid droplet accumulation, suggesting that the standard adipogenic medium is insufficient to ensure successful differentiation (Figure 4).

### 3.4. Effect of PPAR*γ* Stimulation and Nutritional Supplementation on BC ADSC Differentiation Capabilities

**Figure 5:**
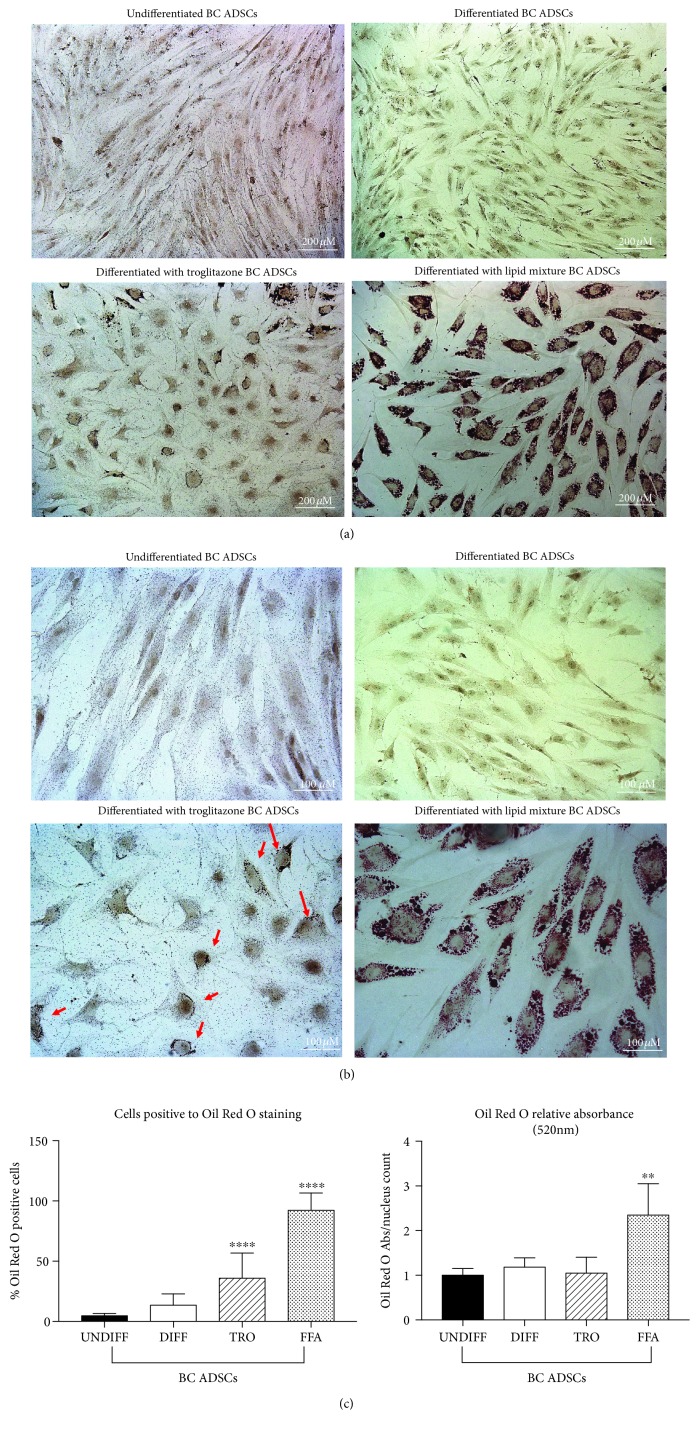
Effect of PPAR*γ* activation and FFA supplementation on BC ADSC differentiation. (a) Adipogenesis was revealed by Oil Red O staining for lipid droplets. (a) shows representative images of BC ADSCs in control conditions and after adipogenic differentiation for 7 days with three different protocols (differentiated BC ADSCs, differentiated BC ADSCs with troglitazone, and differentiated BC ADSCs with lipid mixture). Data are representatives of 5 different isolates. Scale bars: 200 *μ*m. (b) Magnifications of (a) images of BC ADSCs in control conditions and after adipogenic differentiation for 7 days with three different protocols (differentiated BC ADSCs, differentiated BC ADSCs with troglitazone, and differentiated BC ADSCs with lipid mixture). Minimal lipid droplet accumulation after troglitazone supplementation is highlighted by red arrows. Data are representatives of 5 different isolates. Scale bars: 100 *μ*m. (c) Percentage of cells positive to Oil Red O staining and quantification of Oil Red O extracted from lipid droplets measured at 520 nm normalized over nucleus counts. Data are reported as the mean ± SD of 5 different isolates; the statistical significance was determined by one-way ANOVA. ^∗∗∗∗^ *p* < 0.0001 and ^∗∗^ *p* < 0.01 vs. UNDIFF BC ADSCs.

The observation that BC ADSCs seem to present a different and reduced differentiation ability compared to PF ADSCs led us to investigate whether the process can be stimulated by exogenous factors such as situations when the adipogenic differentiation was exacerbated. To do so, we adopted two different differentiation protocols: the culture medium was supplemented in the first protocol with troglitazone (1 *μ*g/mL; TRO), a potent PPAR*γ* activator [36, 37], and in the second one with a lipid mixture of free fatty acids (10% *v*/*v*; FFA), used to mimic a high-fat diet [29]. The rationale is that there could be an altered PPAR*γ* modulation, in line with literature reports indicating an alteration in PPAR*γ* expression in breast cancer cells [20]. Furthermore, we wished to preliminarily identify a potential mechanism for the obesity correlation with breast cancer [25–28]. The accumulation of lipid droplets, investigated by Oil Red O staining, was increased in both conditions, and this is significantly relevant when the lipid mixture was added (Figure 5).

### 3.5. Effect of PPAR*γ* Pharmacologic Inhibition on BC ADSC Differentiation Capabilities

**Figure 6:**
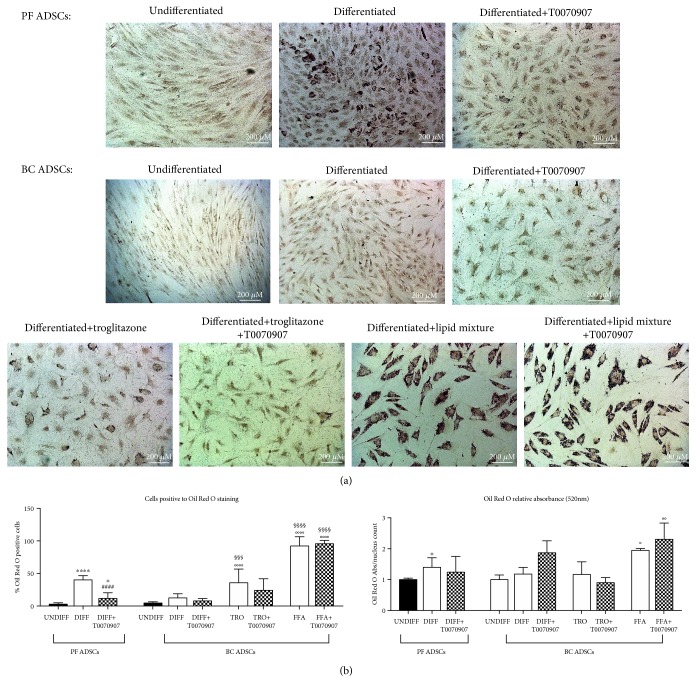
The effect of PPAR*γ* inhibition on PF and BC ADSC differentiation. (a) Adipogenesis was revealed by Oil Red O staining for lipid droplets. (a) shows representative images of PF ADSCs and BC ADSCs in control conditions, after adipogenic differentiation for 7 days and after adipogenic differentiation for 7 days with the PPAR*γ* inhibitor T0070907. Data are representatives of 5 different isolates. Scale bars: 200 *μ*m. (b) Percentage of cells positive to Oil Red O staining and quantification of Oil Red O extracted from lipid droplets measured at 520 nm normalized over nucleus counts. Data are reported as the mean ± SD of 5 different isolates; the statistical significance was determined by one-way ANOVA. ^∗∗∗∗^ *p* < 0.0001 and ^∗∗^ *p* < 0.01 vs. UNDIFF PF ADSCs; ^####^ *p* < 0.0001 vs. DIFF PF ADSCs; °°°°*p* < 0.0001, °°*p* < 0.01, and °*p* < 0.05 vs. UNDIFF BC ADSCs; ^§§§§^ *p* < 0.0001 and ^§§§^ *p* < 0.001 vs. DIFF BC ADSCs.

Following the observation that BC ADSCs seem unresponsive to PPAR*γ* canonical stimulation, we aimed to clarify whether this was true also for the receptor's inhibition. The treatment with the potent and selective PPAR*γ* antagonist T0070907 (1 *μ*M) [38], added to the medium supplemented with troglitazone or FFA, was not able to significantly counteract the lipid droplet accumulation in BC ADSCs (Figure 6 and Supplementary Figure 2). Both the extracted Oil Red O and the percentage of differentiated cells resulted unaffected by the application of T0070907 (Figure 6 and Supplementary Figure 2). As the control of the experiments, T0070907, at the same concentration, was also added to the differentiating medium of PF ADSCs. In this control case, the potent inhibitory effect was appreciable (Figure 6 and Supplementary Figure 2). The demonstration that T0070907 is effective in PF ADSCs but leaves BC ADSCs unaffected again supports the idea that these cells are unresponsive to PPAR*γ* canonical activation.

**Figure 7:**
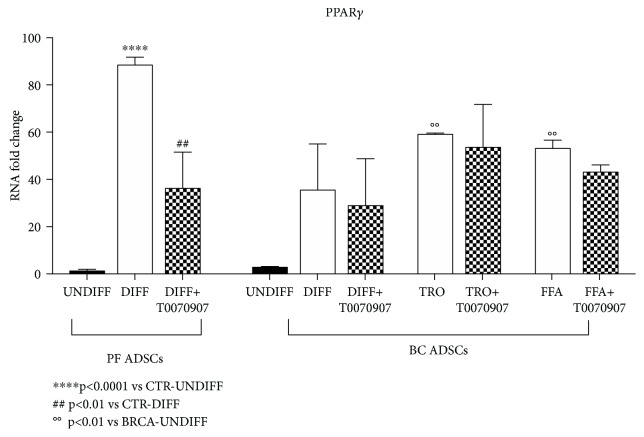
The effect of troglitazone and nutritional supplementation on the expression of PPAR*γ* mRNA. The graph shows BC ADSCs in control conditions and after adipogenic differentiation for 7 days with three different protocols (differentiated BC ADSCs±T0070907, differentiated BC ADSCs with troglitazone±T0070907, and differentiated BC ADSCs with lipid mixture±T0070907). PF ADSCs were inserted as a positive control. The expression of PPAR*γ* mRNA was analysed by real-time PCR. The results are an average of five different isolates, and data are reported as the mean ± SD (*n* = 5). The statistical significance was determined by one-way ANOVA; ^∗∗∗∗^ *p* < 0.001 vs. undifferentiated PF ADSCs; ^##^ *p* < 0.01 vs. undifferentiated PF ADSCs; °°*p* < 0.01 vs. undifferentiated BC ADSCs.

To confirm the evidences reported above, the expression of PPAR*γ* was analysed by real-time PCR in order to verify the molecular changes occurring in the cells during the differentiation process. As expected, in differentiated PF ADSCs, PPAR*γ* mRNA was significantly increased and, conversely, resulted downregulated when cells were also treated with the PPAR*γ* inhibitor (Figure 7). In BC ADSCs, the increase of the adipogenic master gene was observed only when cells were supplemented with troglitazone or FFA (Figure 7). The presence of the inhibitor T007097 did not alter PPAR*γ* expression in BC ADSCs. This suggests that a protocol mimicking nutritional supplementation with a high-fat diet has the ability to modify the genetic profile and differentiation capabilities of BC ADSCs but that this activation is potentially unaffected by PPAR*γ* activity.

## 4. Discussion

This study is aimed at comparing the features of ADSCs isolated from the adipose tissue of breast cancer- (BC-) bearing patients with ADSCs isolated from PF of cancer-free patients, considered here controls. The project stems from various lines of evidences suggesting a role for BC ADSCs during tumour progression and relapse processes. It has been demonstrated that the interactions between cancer and its local microenvironment can determine features such as growth, metastasis, and angiogenesis [11, 12]. Even so, the effects of MSCs on cancer cells are controversial, with evidences indicating the promotion of metastasis and other inhibitions of cancer cell invasion and growth induced by MSCs [42, 43]. The cross-talk between a tumour microenvironment and cancer cells has been characterized both in animal experimental models [10] and in in vitro MDA-MB-231 cells [12], demonstrating that the tumour and its microenvironment create an intertwined loop favouring tumour progression. Mesenchymal stem cells can be regulators of this process, as evidences show their role in promoting breast cancer metastasis [11]. Further, elucidating the cellular features of ADSCs surrounding the tumour microenvironment could be of crucial importance in the modulation of the tumour itself.

We observed that *in vitro* expanded PF and BC ADSCs displayed different characteristics in terms of adipogenic marker expression and phenotypical features of differentiation. This is, to our knowledge, the first study describing the phenotypic characterization of PF and BC ADSCs along with their adipogenic differentiation. Since during differentiation the proliferation of BC ADSCs slowed down shortly with a high rate of mortality (data not shown), 7 days was considered an adequate time to study the process. MSCs are known to show negative expression of hematopoietic surface markers, cluster of differentiation (CD34, CD45, and HLA-DR), and endothelial marker (CD31) but high expressions of CD73, CD90, and CD105 [44]. CD45 is found in hematopoietic cells and regulates cell growth, differentiation, mitotic cycle, and oncogenic transformation [45]. CD90 is a membrane-bound glycoprotein which is expressed by almost 90% of variable tissues, and its function is related to angiogenesis [46]. Consistent with literature reports, almost all of the PF and BC ADSCs express surface markers related to MSCs at very high levels (close to 100%) without any difference depending of the source, while the endothelial and hematopoietic markers were detected only in a small percentage. The slightly lower (but still high) levels of CD90 expression in BC ADSCs compared to PF ADSCs might be caused by the high variability between samples. CD144 (VE-cadherin) is an adhesion protein expressed in endothelial cells. Molecules associated with angiogenesis and vasculogenesis, including this marker, are usually found strongly upregulated in aggressive cancers [47, 48], consistent with our observations. CD146, a typical adhesion marker, is reported as a common surface marker of MSCs, and the activation of CD146 induces the dynamic process of dimerization in response to stimuli in a tumour microenvironment [49]. Indeed, the biological significance of CD146 in a normal tissue remains unclear, even if it has been suggested to have a fundamental role in cancer, angiogenesis, and cardiovascular diseases [50]. It has been reported that the surface markers such as CD133, in combination with CD29/CD24 and CD44/CD166, may correlate with high Wnt activity and identify stem cells in various tumour types and their possible interaction with the microenvironment [51]. Interestingly, in BC ADSCs, the markers CD144, CD146, CD166, and KDR result significantly deregulated suggesting oncogenic alteration also in the tumour microenvironment with a possible role in favouring angiogenesis, tumour cell migration, and proliferation [52].

Adipogenic differentiation is regulated by a complex network of transcription factors. It begins with increased expressions of CCAT/enhancer-binding protein (C/EBP), which in turn activate peroxisome proliferator-activated receptor *γ* (PPAR*γ*) [53]. In mammalian cells, the PPAR*γ* and the C/EBPs such as C/EBP*α*, C/EBP*β*, and C/EBP*δ* are considered the key early regulators of adipogenesis, while fatty acid-binding protein 4 (FABP4), adiponectin, and fatty acid synthase (FAS) are responsible for the formation of mature adipocytes [54]. It is reported that FABP4 is an adipokine with a distinct role of transcriptional and metabolic regulation in ASCs [55]. FABP4 is increased during the differentiation only in PF ADSCs, and interestingly, its expression decreases when BC ADSCs are induced to differentiate. PF ADSCs show a significant increase of PPAR*γ* during the adipogenic differentiation, in accordance with other works stating that PPAR*γ* is directly associated with lipid metabolism. On the other hand, in BC ADSCs induced to differentiate, PPAR*γ* does not show a significant difference with the undifferentiated cells. This is confirmed with multiple techniques, observing a phenotypic (immunohistochemistry) and genetic (real-time PCR) incapability of these cells to express the PPAR*γ* receptor and thus efficiently differentiate. Indeed, BC ADSCs show an impaired ability compared to PF ADSCs to differentiate into adipocytes. At this stage of differentiation, no differences were observed for C/EBP*β* while C/EBP*δ* was increased in both cellular populations, as expected.

The observations indicating an impaired differentiation ability of BC ADSCs, together with literature reports describing PPAR*γ* deregulation in breast cancer patients and its possible role as a therapeutic target [20–23], lead us to further investigate the role that this receptor could play in BC ADSC differentiation. To this end, we attempted to stimulate BC ADSC differentiation with the supplementation of the PPAR*γ* activator troglitazone [37]. We found that this slightly increases BC ADSC adipogenic differentiation but that this increase is not impaired following the supplementation with the PPAR*γ* inhibitor T007097, supporting the claim that BC ADSCs are partially insensitive to PPAR*γ* modulation. We hypothesise that troglitazone could also mediate PPAR*γ*-independent effects on BC ADSCs but the identification of a specific activated pathway would require further analysis. Interestingly, previous literature reports demonstrate that troglitazone inhibited telomerase activity in MDA-MB-231 cells, independently of PPAR*γ* activity [56].

Moreover, we decided to investigate the concept that nutrients and obesity may have in BC progression [9, 16], evaluating the effects of a high-fat diet on the differentiation properties of BC ADSCs. The aim was to gain more in depth insight into epidemiologic and experimental evidences suggesting the role of nutritional health and high/low-fat diets in the regulation of breast cancer prognosis [16, 24–28]. The results obtained were very interesting, demonstrating a significant lipid accumulation and also a significant increase in PPAR*γ* expression. This observation could support the evidences that nutrition influences the breast cancer microenvironment, and targeting this pathway may be of key importance in modulating tumour progression. In the experimental context of BC ADSCs, the PPAR*γ* inhibitor T007097 was proved inefficient in modulating BC ADSC adipogenesis after lipid mixture supplementation.

Together, these results indicate that BF ADSCs are unable to differentiate when stimulated with a standard protocol, but this can be partially overcome by supplementing the differentiation medium with troglitazone or FFA. The demonstration that BC ADSCs were proved unresponsive to the PPAR*γ* inhibitor T007097 further supports the hypothesis that this receptorial signalling is partially impaired in these cells, although further experimental analyses are required to elucidate the specific intracellular pathways activated.

## 5. Conclusions

In conclusion, this study shows that there is a significant difference between healthy fat tissue and fat tissue of BC-bearing patients when observing molecular marker expression, phenotype, and adipogenic differentiation capability. Indeed, BC ADSCs present with an impaired differentiation efficiency. The present study also shows some preliminary, although very interesting, results correlating the role of nutrition on a tumourigenic microenvironment, but more studies are necessary to elucidate the precise mechanisms. Furthermore, the observation of BC ADSC unresponsiveness to PPAR*γ* canonical stimulation and inhibition could provide evidence of new deregulated and targetable cancer pathways. This is the first study that characterizes ADSCs from fat tissue near the breast cancer and compares them with ADSCs derived from periumbilical fat. Furthermore, for the first time, the adipogenic differentiation potential of BC ADSCs was studied. With these promising results, further studies aiming at clarifying the mechanism of adipogenic differentiation from the BC microenvironment and its role in BC development could be of crucial importance in the understanding of breast cancer progression and metastasis.

## Figures and Tables

**Table 1:** Clinical characteristics of the 20 breast cancer-affected patients enrolled in the present study.

*N*	Age	Histology	Grading	ER	PR	Ki67 (%)	Her2/neu	TNM	Stage	Operation
1	56	LIC	G2	+	+	10	-	pT1b, N0(sn)	IA	QDR+SNB
2	69	DIC	G2	+	+	25	-	pT1b, Nx	IA	QDR
3	75	DIC	G2	+	-	8	+	pT1c, N1mi(sn)	IB	QDR+SNB
4	82	DIC	G3	+	-	35	+	pT2, pN1a	IIB	QDR+AND
5	54	DIC	G3	+	-	25	+	pT1c, pN0(sn)	IA	QDR+SNB
6	48	DIC	G2	+	+	40	+	pT2, N1a	IIB	QDR+AND
7	63	DIC	G3	+	+	25	+	pT1c, N0(sn)	IA	QDR+SNB
8	66	DIC	G2	+	+	10	-	pT1b, N0(sn)	IA	QDR+SNB
9	64	DIC	G2	+	+	8	+	pT1c, pN0(sn)	IA	QDR+SNB
10	78	DIC	G2	+	+	12	+	pT1c, pN0(sn)	IA	QDR+SNB
11	47	DIC^∗^	G2	+	+	8	+	pT1c, pN1mi	IB	QDR+SNB
12	69	DIC	G2	+	+	15	-	pT1c, pN0(sn)	IA	QDR+SNB
13	44	DIC	G1	+	+	12	+	pT1c, pN1mi	IB	QDR+SNB
14	52	DIC	G3	-	-	85	-	pT1c, pN1a	IIA	QDR+AND
15	66	DIC	G2	+	+	18	+	pT2, pN0(sn)	IIA	QDR+SNB
16	58	LIC	G2	+	+	10	-	pT2, pN3a	IIIC	QDR+AND
17	68	DIC	G1	+	+	5	+	pT1b, pN0(sn)	IA	QDR+SNB
18	76	DIC	G3	+	+	55	+	pT2, pN1a(sn)	IIB	QDR+AND
19	65	DIC	G2	+	+	12	+	pT1b, pN0(sn)	IA	QDR+SNB
20	63	LIC	G2	+	+	10	+	pT2, pN1mi(sn)	IIB	QDR+SNB

ER: estrogen receptors; PR: progesterone receptors; TNM: according to AJCC (American Joint Committee on Cancer)/UICC (Union for International Cancer Control) classification: release 2017; LIC: lobular infiltrating carcinoma; DIC: ductal infiltrating carcinoma; DIC^∗^: ductal infiltrating carcinoma, papillary variant; QDR: standard quadrantectomy; SNB: sentinel node biopsy; AND: axillary node dissection; sn: sentinel node; mi: node micrometastasis.

## Data Availability

Primary cultures were obtained from PF and from mammary fat, isolated from patients at University San Paolo Hospital of Milan (Milan, Italy). All data are provided in full in results of our manuscript, and the necessary detail can be provided by the corresponding authors under request.

## Supplementary Materials

Supplementary Materials Supplementary Table 1: list of primer sequences. Supplementary Figure 1: cell surface phenotype of 20 BC ADSCs (red boxes) and 8 PF ADSCs (black boxes). All percentages were obtained by flow cytometry analysis. Results were obtained from three independent experiments. The statistical significance was determined by Student's *t*-test; ^∗∗∗∗^ *p* < 0.0001, ^∗∗∗^ *p* < 0.001, ^∗∗^ *p* < 0.01, and ^∗^ *p* < 0.05 vs. PF ADSCs. Supplementary Figure 2: adipogenesis was revealed by Oil Red O staining for lipid droplets. The panel shows image magnifications of Figure 6(a), showing PF ADSCs and BC ADSCs in control conditions and after adipogenic differentiation for 7 days ± PPAR*γ* inhibitor T0070907. Data are representatives of 5 different isolates. Scale bars: 100 *μ*m.
