# Supplementary material for: Adipose-Derived Stem Cells from Fat Tissue of Breast Cancer Microenvironment Present Altered Adipogenic Differentiation Capabilities
Source: Stem Cells Int. 2019 Aug 14;2019:1480314. doi: 10.1155/2019/1480314 (PMC6710814; doi:10.1155/2019/1480314)

Supplementary Materials

Supplementary Table 1. List of primer sequences

PPARγ-Fw: CAAGAGTACCAAAGTGCAATCAAAGTGGAG
PPARγ-Rev: GTTCTCCGGAAGAAACCCTTGCATCCTTCA
GAPDH-Fw: CTTTTGCGTCGCCAG
GAPDH-Rev: TTGATGGCAACAATATCCAC

Supplementary Figure 1. Cell surface phenotype of 20 BC ADSCs (red boxes) and 8 PF ADSCs (black boxes). All percentages were obtained by flow cytometry analysis. Results were obtained from three independent experiments. The statistical significance was determined by Student's t-test; ****$p<0.0001$, ***$p<0.001$, **$p<0.01$ and *$p<0.05$ vs PF ADSCs.

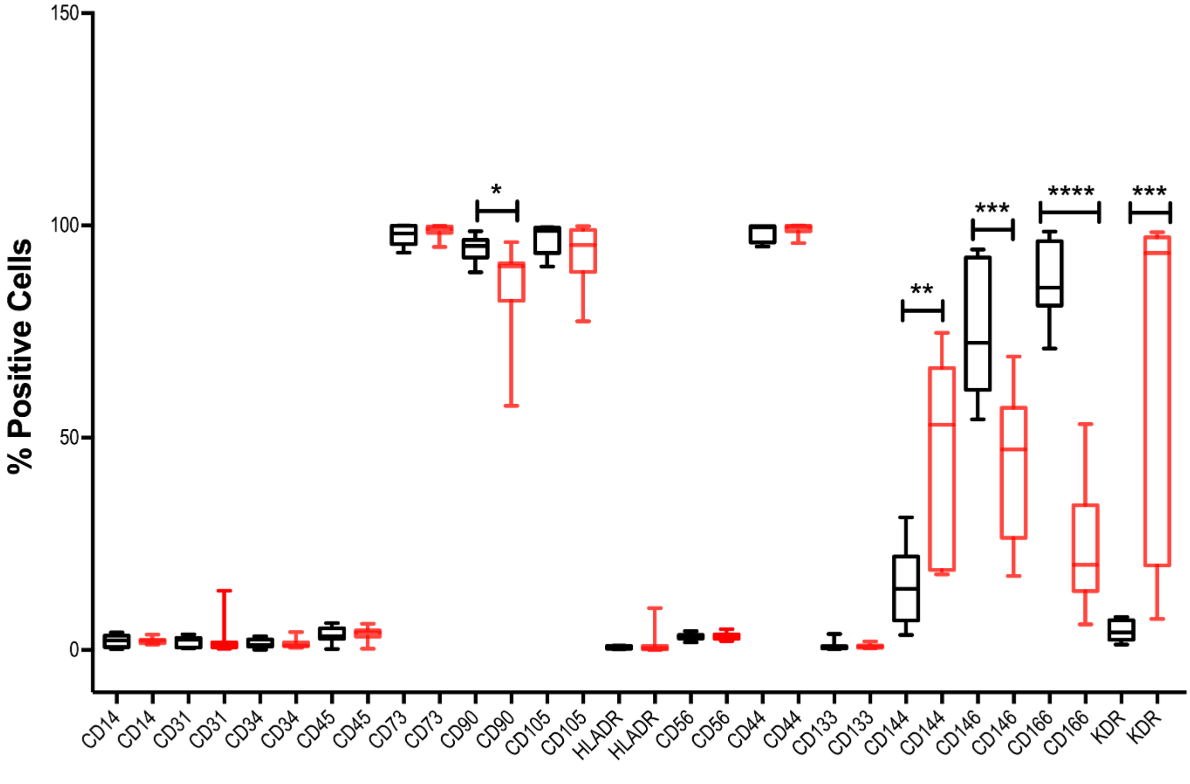

Supplementary Figure 2. Adipogenesis was revealed by Oil Red O staining for lipid droplets. The panel shows image magnifications of Figure 6 Panel A, showing PF ADSCs and BC ADSCs in control conditions and after adipogenic differentiation for 7 days ± PPARγ inhibitor T0070907. Data are representative of 5 different isolates. Scale bars: 100 μm

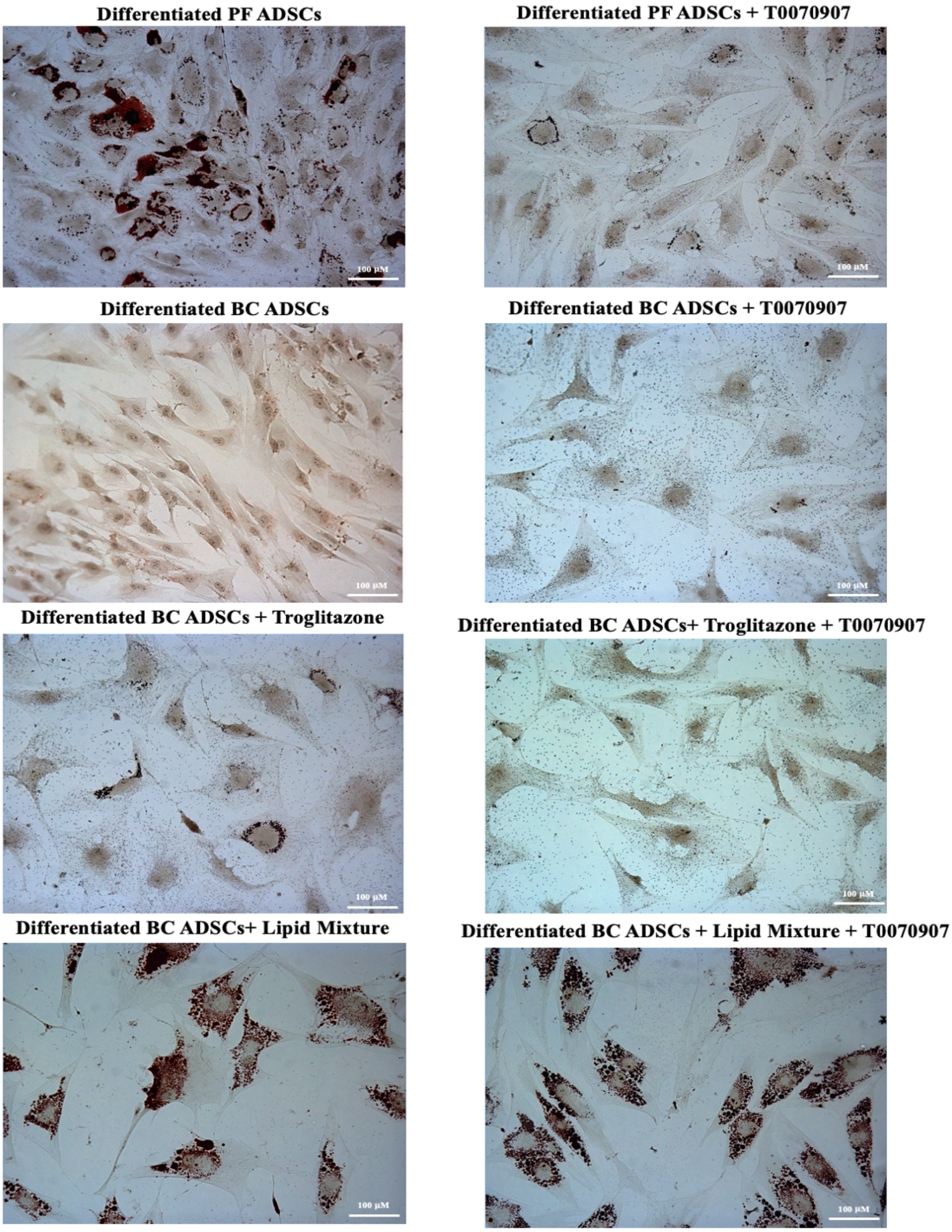

Supplement: Supplementary Materials — Supplementary Table 1: list of primer sequences. Supplementary Figure 1: cell surface phenotype of 20 BC ADSCs (red boxes) and 8 PF ADSCs (black boxes). All percentages were obtained by flow cytometry analysis. Results were obtained from three independent experiments. The statistical significance was determined by Student's t-test; ∗∗∗∗ p < 0.0001, ∗∗∗ p < 0.001, ∗∗ p < 0.01, and ∗ p < 0.05 vs. PF ADSCs. Supplementary Figure 2: adipogenesis was revealed by Oil Red O staining for lipid droplets. The panel shows image magnifications of Figure 6(a), showing PF ADSCs and BC ADSCs in control conditions and after adipogenic differentiation for 7 days ± PPARγ inhibitor T0070907. Data are representatives of 5 different isolates. Scale bars: 100 μm. [file 1480314.f1.pdf]
